# Naturally occurring hybrids of coral reef butterflyfishes have similar fitness compared to parental species

**DOI:** 10.1371/journal.pone.0173212

**Published:** 2017-03-03

**Authors:** Stefano R. Montanari, Jean-Paul A. Hobbs, Morgan S. Pratchett, Line K. Bay, Lynne van Herwerden

**Affiliations:** 1 AIMS@JCU, Australian Institute of Marine Science, College of Science and Engineering, James Cook University of North Queensland, Townsville, QLD, Australia; 2 Department of Environment and Agriculture, Curtin University, Bentley, WA, Australia; 3 ARC Centre of Excellence for Coral Reef Studies, James Cook University of North Queensland, Townsville, QLD, Australia; 4 Australian Institute of Marine Science, Townsville, QLD, Australia; 5 College of Science and Engineering, James Cook University of North Queensland, Townsville, QLD, Australia; 6 Centre for Comparative Genomics, James Cook University of North Queensland, Townsville, QLD, Australia; University of California Santa Cruz, UNITED STATES

## Abstract

Hybridisation can produce evolutionary novelty by increasing fitness and adaptive capacity. Heterosis, or hybrid vigour, has been documented in many plant and animal taxa, and is a notable consequence of hybridisation that has been exploited for decades in agriculture and aquaculture. On the contrary, loss of fitness in naturally occurring hybrid taxa has been observed in many cases. This can have negative consequences for the parental species involved (wasted reproductive effort), and has raised concerns for species conservation. This study evaluates the relative fitness of previously documented butterflyfish hybrids of the genus *Chaetodon* from the Indo-Pacific suture zone at Christmas Island. Histological examination confirmed the reproductive viability of *Chaetodon* hybrids. Examination of liver lipid content showed that hybrid body condition was not significantly different from parent species body condition. Lastly, size at age data revealed no difference in growth rates and asymptotic length between hybrids and parent species. Based on the traits measured in this study, naturally occurring hybrids of *Chaetodon* butterflyfishes have similar fitness to their parental species, and are unlikely to supplant parental species under current environmental conditions at the suture zone. However, given sufficient fitness and ongoing genetic exchange between the respective parental species, hybrids are likely to persist within the suture zone.

## Introduction

Natural hybridisation was once considered rare and unimportant [1], but a large and increasing body of literature suggests that this process may be critically important for both adaptation and speciation [2]. Importantly, natural hybridisation can play a role in the formation of new species if it produces novel genotypes that outperform their parental species or persist in previously unoccupied niches [3]. Conversely, hybridisation can contribute to the loss of biodiversity through extinction [4] or reverse speciation [5, 6]. The evolutionary consequences and implications of hybridisation are largely dependent upon the extent to which hybrids interact with their parent species (e.g., differential habitat use, assortative mating) and individual fitness.

Heterosis (commonly referred to as hybrid vigour) [7] is a notable consequence of hybridisation and has been exploited for decades in agriculture and aquaculture. Hybrids of many plant and animal species exhibit increased vigour (e.g., faster growth, larger size, and higher reproductive output) and can be more stress tolerant relative to either parental species [8, 9]. However, the mechanistic underpinnings of heterosis are only just beginning to emerge, and involve the complex interplay of epigenetic modification of gene regulation [9] and environmental selection for novel genotypes [10]. In at least some instances, hybrid genotypes experience marked loss of fitness relative to their parental species, which is commonly attributed to meiotic irregularities or genetic incompatibility [10]. In the extreme, hybrids may be sterile or non-viable [11]. However, the fitness of hybrids is influenced by both endogenous (environment-independent) and exogenous (environment-specific) selective processes [10]. Where genetic incompatibility is not an issue [2], exogenous selection enables hybrid genotypes to outperform their parental counterparts in at least some situations and environments [10].

Natural hybridisation has been particularly well studied among terrestrial and freshwater species [12–15]. Herein, the prevalence of hybridisation (largely apparent from genetic analyses that reveal high levels of introgression) shows that postzygotic barriers to inter-breeding among recently diverged species are rarely complete, but may be permeable in time or space [2]. Hybridisation can therefore provide an additional (and potentially major) source of genetic variation, contributing to adaptive radiation in highly diverse or changing environments [16, 17]. Recent pulses in the incidence of “natural” hybridisation are widely attributed to anthropogenic degradation or disruption of natural ecosystems, such as translocation of species and fragmentation of habitats [18, 19]. Hybridisation among some wild species would not have occurred naturally and is leading to extensive genetic mixing and effective extinction of one or both parental species [18]. However, genetic variation through hybridisation may also yield novel genotypes and expedite adaptation, thereby ensuring species persistence in the face of changing environmental conditions [20, 21].

The prevalence and importance of hybridisation has not been appreciated in marine systems until very recently [22, 23]. Given the very high diversity and relatively recent divergence of species in some marine habitats (e.g., coral reefs), it is little surprise that hybridisation is highly prevalent among marine species [24–27]. Hybridisation is particularly apparent in narrow and specific geographic areas, where regional biotas intersect at biogeographic borders or suture zones [28–30]. As shown in other ecosystems, taxonomic bias in the occurrence of hybridisation is also evident among marine species: hybridisation is particularly prevalent among coral reef fishes, especially butterflyfishes (family Chaetodontidae) and angelfishes (family Pomacanthidae) [27, 29, 31–33]. Accordingly, there has been disproportionate research attention given to the molecular and ecological factors that promote hybridisation in these groups [26, 34–38]. However, the evolutionary implications of hybridisation in coral reef fishes are not yet well understood.

The purpose of this study was to explicitly test for variation in fitness of documented hybrids relative to parental species for coral reef butterflyfishes (*Chaetodon*: Chaetodontidae). Fitness is ultimately a measure of individual reproductive success and is the average contribution to the next generation gene pool by individuals of a particular genotype. Directly measuring fish reproductive success in the wild can prove impractical in the absence of long-term mark-and-recapture studies coupled with parentage analysis. In the case of *Chaetodon* hybrids, fertility has been either anecdotally reported or inferred through the detection of introgression [37, 38]. Some differences in growth rates and longevity have been reported in one other case of tropical reef fish hybridisation: *Cephalopholis* groupers at Christmas and Cocos (Keeling) Islands [39]. Further, increased growth rates, particularly during early life-history stages, are associated with enhanced survivorship, faster maturation, and greater female fecundity at a given age, thereby representing a useful proxy for fitness [40]. The aims of this paper were to compare fitness between parental species and naturally occurring butterflyfish hybrids of genus *Chaetodon* based on: 1) reproductive output, measured as relative gonad mass; 2) body condition, inferred from hepatocyte vacuolation; and 3) growth, inferred from size at age relationships.

## Materials and methods

### Study sites and species

Sampling was conducted between July 2008 and November 2013 at Christmas Island, Australia (10.4475° S, 105.6904° E). All samples used in the fertility and body condition analyses described below were collected over 2 weeks between November 15^th^, 2013 and November 28^th^, 2013, in order to minimise differences due to yearly or seasonal variation (Table 1). The study was undertaken in accordance with the Committee of Animal Ethics of James Cook University of North Queensland (AEC Approval Number: A1757). All fishes were speared on SCUBA and immediately euthanized by severing the first postcranial trunk vertebra, in accordance with the permit above. This study focussed on two hybridising butterflyfish groups, for which detailed genetic analyses have confirmed the status of hybrids and parental species [37]. Despite some between-group differences in mitochondrial inheritance and introgression rates, hybridisation appears to be on going in both groups, and the hybrids display no obvious differences in ecology or behaviour relative to their parental species [37]. To date however, nothing is known about the fitness of these hybrids and whether they are likely to persist in the wild. Total length (TL) was measured to the closest mm and each fish was weighed (after blotting) on electronic scales to the closest mg. Livers and gonads were extracted and weighed to the closest mg, and stored in 4% buffered formaldehyde for histological examination. Otoliths were extracted, rinsed in ethanol and preserved dry for size at age analysis.

**Table 1:** Sample sizes for the components of the present study, divided by taxon.

Taxon	Fertility	Body condition	Size at age
*Chaetodon guttatissimus*	29	14	87
*C*. *punctatofasciatus*	12	12	31
*C*. *guttatissimus* × *punctatofasciatus* hybrids	15	10	37
*C*. *trifasciatus*	4	3	39
*C*. *lunulatus*	5	4	23
*C*. *trifasciatus* × *lunulatus* hybrids	3	3	13

### Fitness measurements

#### Fertility

To confirm that hybrid fishes were fertile, we undertook a qualitative histological assessment of female and male gonads for all taxa. Preserved gonads were processed using an automatic tissue processor (Intelsint–EFTP) with ascending grades of ethanol, three changes of absolute ethanol, and cleared in xylene followed by three changes of paraplast wax. Tissues were then embedded using a Shandon Histocentre 3 embedding centre, and blocks were cut at 5μm using a Micron rotary microtome. Slides were dried at 60°C, then manually stained with Mayer’s Haematoxylin and Young’s eosin/erythrosine, and mounted in DPX [41]. Each slide was viewed under transmitted light with a compound microscope, and three haphazardly chosen sections photographed at 400x using an Olympus DP21 system to provide evidence of hybrid fertility (e.g. presence of gametocytes). Further, relative gonadal mass, or gonadosomatic index (GSI) [42], was calculated for all individuals in each taxon, and used as a proxy for reproductive output. Fishes used in these analyses were all paired at the time of collection, indicating they had reached sexual maturity [43]. Butterflyfish are thought to spawn year-round under ideal conditions [44] and the assumption that all specimens were reproductively synchronised, with similarly developed gonads, was deemed reasonable. The *C*. *trifasciatus* hybridising group was data deficient, and therefore not included in formal statistical comparisons. For the *C*. *guttatissimus* group, one-way analysis of variance (ANOVA) was used to evaluate the effect of taxon on GSI, separately for each gender.

#### Body condition

To provide a measure of general body condition, livers were prepared for histological examination following the methods described above for gonads. Hepatocyte vacuolation was used as a proxy for liver lipid content and body condition [45, 46]. We recorded the proportion of 42 points that intercepted vacuolated hepatocytes [47] using a grid superimposed on each photograph in ImageJ [48]. Generalised linear models assuming a binomial distribution [49] were used, for each hybrid group separately, to determine the effect of taxon on hepatocyte vacuolation.

#### Aging

To determine the age of specimens, sagittal otoliths were embedded in an epoxy resin block and a transverse section (approximately 400 μm) was cut from each using a Buehler low-speed saw to expose the otolith core [50]. Individual sections were mounted on glass microscope slides with thermoplastic cement and polished with 1200-grit wet-dry sanding paper [50]. Each section was viewed under transmitted light with a dissecting microscope for annual increments and a compound microscope for daily increments. Where possible, the number of presumed daily or annual increments was counted along the dorsal axis, as the increments were generally more distinct in this region.

#### Size at age

Von Bertalanffy growth functions (VBGFs) [51] were fitted to length at age data, separately for each taxon. Unconstrained least-squares estimates of the VBGF parameters *L*_*∞*_ (asymptotic length), *K* (growth rate) and *t*_*0*_ (theoretical time at length 0) were generated using R function *nls* [52]. The effect of taxon on VBGFs was determined by assessing the degree of overlap of the 95% confidence intervals around the VBGF parameter estimates.

## Results and discussion

### Fertility

**Fig 1:**
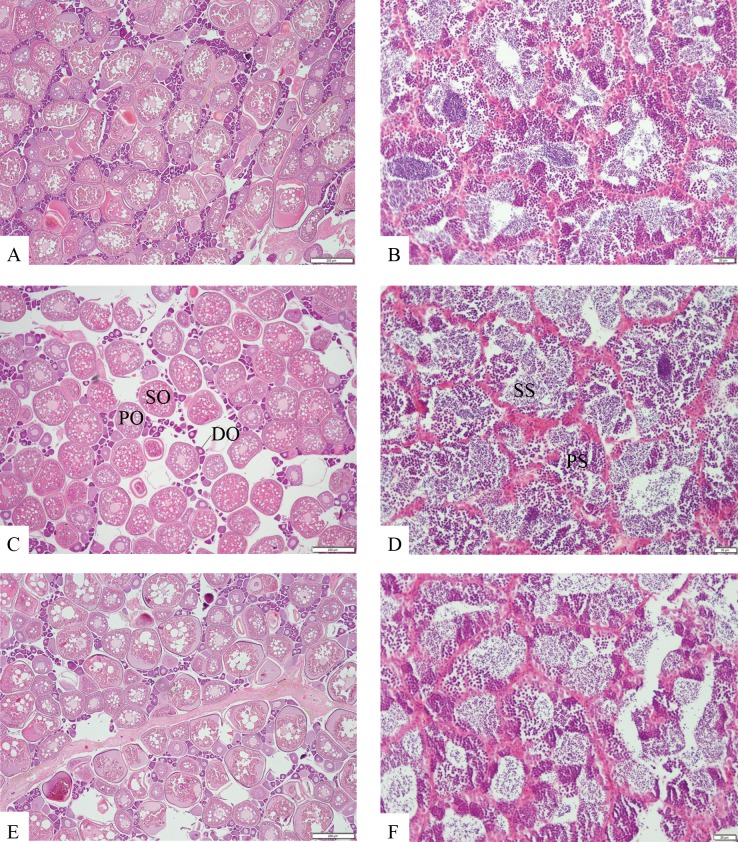
Female and male gonads of hybridising *Chaetodon* butterflyfishes. Typical appearance of female (A, C and E) and male (B, D and F) gonads of hybridising *Chaetodon* butterflyfishes from the Christmas Island suture zone. *Chaetodon guttatissimus* (A and B); *C*. *guttatissimus × C*. *punctatofasciatus* hybrids (C and D); *C*. *punctatofasciatus* (E and F). Mature hybrids (C and D) of both sexes had normal gametocytes, similar to those of their parental species, at all stages of development. DO: primary oocyte in diplotene stage; PO: primary oocyte; SO: secondary oocyte; PS: primary spermatocyte; SS: secondary spermatocyte.

**Fig 2:**
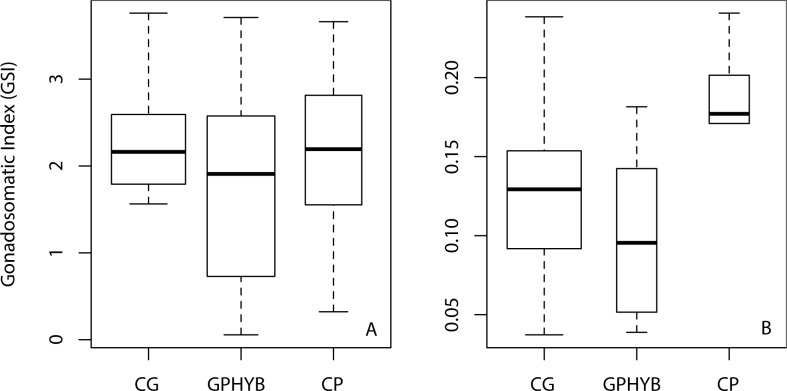
Gonadosomatic indices of the *C*. *guttatissimus* hybrid group at Christmas Island. The width of boxes is proportional to the square root of sample size (see Table 1), for females (A) and males (B). CG: *C*. *guttatissimus*; GPHYB: *C*. *guttatissimus × C*. *punctatofasciatus* hybrids; CP: *C*. *punctatofasciatus*.

Mature hybrid females and males had normally developed gonads, similar to those of the parental species, showing all stages of oocyte and spermatocyte development respectively (Fig 1). GSI did not vary significantly between hybrids and parental species in either females or males of the *C*. *guttatissimus* group (Fig 2). Differences in GSI between sexes were clear in all taxa and variation around the median was high for all sex/taxon combinations (Fig 2). GSIs of hybrid females and males were no different to those of their parent species of the same sex (F_(2,26)_ = 0.59, p = 0.56 and F_(2,24)_ = 0.88, p = 0.43), respectively (Fig 2).

### Body condition

**Fig 3:**
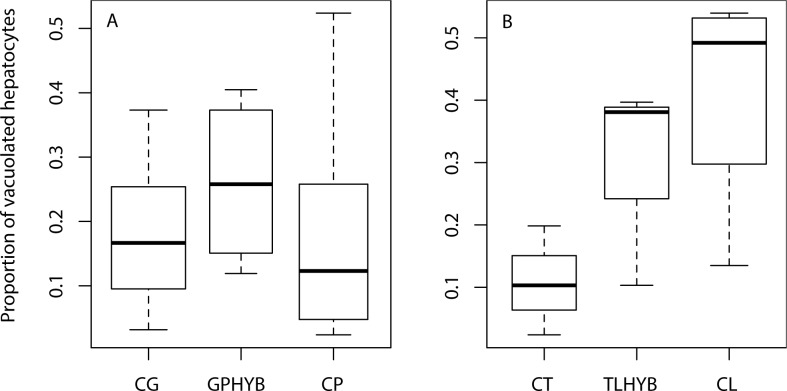
**Hepatocyte vacuolation in *C*. *guttatissimus* (A) and *C*. *trifasciatus* (B) hybrid groups.** Solid boxes indicate standard errors and whiskers indicate range (see Table 1 for sample sizes). CG: *C*. *guttatissimus*; GPHYB: *C*. *guttatissimus × C*. *punctatofasciatus* hybrids; CP: *C*. *punctatofasciatus*; CT: *C*. *trifasciatus*; TLHYB: *C*. *trifasciatus × C*. *lunulatus* hybrids; CL: *C*. *lunulatus*.

Hepatocyte vacuolation was not influenced by taxon in either hybrid group (Fig 3). In both groups, within-taxon variability in liver lipid content was high (Fig 3). In the *C*. *guttatissimus* group, median hepatocyte vacuolation was generally low and ranged from 12% to 26% (Fig 3A). Hybrid *C*. *guttatissimus × C*. *punctatofasciatus* had similar levels of liver lipids compared to their parent species (z_(33)_ = 0.50, p = 0.62). In the *C*. *trifasciatus* group, median hepatocyte vacuolation had a broader range from 10% to 48% (Fig 3B) and hybrids were not significantly different from their parental species (z_(7)_ = 0.55, p = 0.58) from the suture zone, potentially confounded by small sample size.

### Size at age

**Fig 4:**
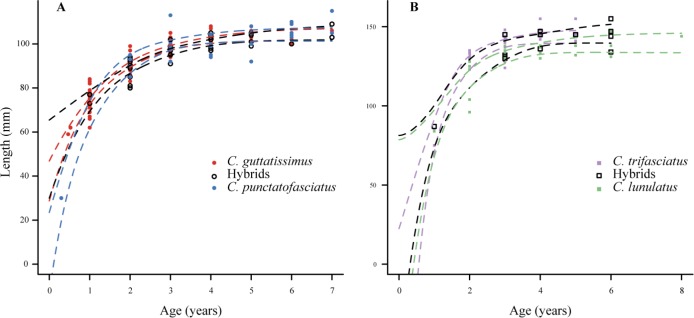
Size at age relationships in hybridising *Chaetodon* butterflyfishes at Christmas Island. Von Bertalanffy growth functions fitted to size at age data of all taxa in the *C*. *guttatissimus* (A) and *C*. *trifasciatus* (B) hybrid groups. Dots are individual data points and dashed lines are 95% confidence intervals around the fitted models. For sample sizes refer to Table 1.

There was no difference in asymptotic length for parental versus hybrid individuals in either species group (Fig 4). Average L_∞_ estimates were consistent with observed maximum lengths: *C*. *guttatissimus* 104.66 mm, *C*. *guttatissimus × C*. *punctatofasciatus* hybrids 105.71 mm, *C*. *punctatofasciatus* 104.41 mm, *C*. *trifasciatus* 139.79 mm, *C*. *trifasciatus × C*. *lunulatus* hybrids 146.52 mm and *C*. *lunulatus* 143.91 mm. The 95% confidence intervals of estimates showed a high degree of overlap between parent species and hybrids in both groups (Fig 4). This suggests marginal differences in asymptotic length (L_∞_), growth rate (K) and theoretical time at length 0 (t_0_), between hybrids and parental species in each respective group. This indicates that hybrid taxa in both groups grow at a similar rate to their parent species within the suture zone.

This study indicates that inter-specific breeding across two distinct species groups of *Chaetodon* butterflyfishes results in viable hybrid offspring. Naturally occurring hybrids of *Chaetodon* butterflyfishes considered here (*C*. *guttatissimus × C*. *punctatofasciatus* and *C*. *trifasciatus × C*. *lunulatus*) have similar condition to their respective parental species from the suture zone in at least three distinct fitness related traits including fecundity, body condition, and growth. Heterosis or decreased fitness have been documented in some hybrid teleost fishes (e.g. salmonids, minnows, barramundi) [53–55] and Payet, Hobbs (39) found some possible differences in longevity and growth in hybrid groupers. Here we explicitly test for increased vigour following interspecific breeding of wild tropical reef fishes, by examining several fitness-associated traits.

Although hybrid butterflyfishes examined here exhibited similar levels of fecundity (GSI), body condition (hepatocyte vacuolation), and growth (size at age) compared to parental species from the suture zone, it is possible that heterosis or decreased fitness may be expressed in other traits or environments not evaluated here. Importantly, hybrids of some freshwater fishes (e.g. hybrids of pupfish and minnow, cichlids) exhibit enhanced performance and/or capacity to exploit novel niches that are generally unavailable to parental species [17, 55, 56]. Ecological surveys for the *Chaetodon* species groups considered in this study show that hybrids occupy the same habitats and ostensibly use the same resources as their parent species [37, 38]. This is not unexpected, given that hybridising species of *Chaetodon* butterflyfishes tend to exhibit striking similarities in their ecology [29], which may well be an important requisite for hybridisation between teleost fishes [26]. Hybrids may nonetheless have traits that differentiate them from their parental species, and enable increased tolerance of changing environmental conditions or increased occupation of distinct niches not detected here. This would only be apparent from either ongoing monitoring of hybrid prevalence in the field or experimental tests of physiological tolerances.

This study represents a snapshot in time and space of the relative fitness of hybrids and their respective parent species, providing an important reference point. Ongoing monitoring of hybrid prevalence is important, because if hybrids disperse away from the Christmas Island suture zone they may encounter different environmental conditions. It is unknown what the relative fitness of the hybrids would be in these new environments, but hybrid freshwater fishes have been successful in exploiting new environments [17, 56]. In addition, environmental conditions are changing throughout all oceans and reefs—including those at Christmas Island [57]—for a number of reasons, thus the fitness of hybrids compared to parental species may change at the suture zone in the future. For example, rising sea temperatures directly impact reef fish metabolism [58, 59] and indirectly impact corallivorous species (such as the butterflyfishes in this study) through thermal bleaching and mortality of corals that are important for food and habitat [60–63]. Finally, given that hybrids represent a continual source of novel genetic combinations, the ongoing hybridisation of butterflyfishes at Christmas Island may, in the future, produce hybrids that are fitter than their parent species [20]. In this study we found hybrids that had similar fitness related traits to parent species at the time of collection at Christmas Island. Further research on these taxa at other times and locations will provide insights into how the relative fitness of hybrids changes with environmental conditions.

Apparent similarities in trait values for hybrid versus parental species of *Chaetodon* butterflyfishes may partly reflect the limited sample sizes, especially in terms of numbers of hybrids sampled (n = 3–13 for *C*. *trifasciatus × C*. *lunulatus* and n = 10–37 for *C*. *guttatissimus × C*. *punctatofasciatus*, see also Table 1). Unfortunately, limited sample sizes are an inherent limitation for studies of natural hybridisation, because these taxa are often rare [64]. The *C*. *guttatissimus* group was analysed with a minimum of ten hybrid individuals and showed the same patterns as the *C*. *trifasciatus* group with a minimum of 3 hybrids. We would expect discrepancy in results between groups if small sample sizes played a major role.

The vigour expressed in some F1 hybrids is often lost in subsequent generations (F2 and/or backcrosses) [54]. Distinguishing between pure individuals and later generation backcrosses (F4 or later) can represent a challenge and may not be particularly useful, because the signal of hybridisation is lost [65]. Further, the limited sample size did not allow for the subdivision of individuals into discrete hybrid classes (e.g F1, backcrosses) for the statistical analyses presented here. Both species groups examined here exhibited the full spectrum of hybrid genotypes (e.g. F1, F2 and backcrosses), as indicated by microsatellite data in previous studies [37] and subsequently confirmed with whole genome SNP scans (unpublished data). These observations *per se* confirm not only the fertility, but also the viability of *Chaetodon* hybrids, and are corroborated by the histology and GSI data presented here. Hybrids in both groups backcross with either parent species, in frequencies directly proportional to their relative abundance (i.e. non-assortatively) [37]. They are also infrequently seen in hybrid-hybrid pairs, suggesting that the production of F2 individuals is a distinct possibility, as evident from genetic analyses [37]. Indeed, F1 individuals are the least common in both groups [37] and hence represent the minority of the hybrids sampled in this study. It seems therefore reasonable to conclude that the loss of fitness frequently reported in subsequent generation hybrids [66, 67] does not apply to butterflyfishes of genus *Chaetodon* at Christmas Island, where they hybridise naturally.

## Conclusions

Hybridisation can play an active role in shaping populations and communities, thus impacting biodiversity. One or both parent species in the two *Chaetodon* groups considered here are locally rare [37, 38, 68]. Hybridisation can be an evolutionarily relevant source of genetic diversity for these species, because the probability of conspecific mating is low [17]. Unlike cases of hybridisation that have anthropogenic causes and consequences that are deemed detrimental to the species involved [6, 19], hybridisation among *Chaetodon* butterflyfishes and other coral reef fishes at Christmas Island [28] seems to find its roots in secondary contact between recently diverged sister species [37, 38]. The similarity in fitness related traits between butterflyfish hybrids and their parental species supports the likely persistence of hybrids and their potential as sources of novel genetic diversity, adaptability and biodiversity within this isolated geographical location.
